# Association Between Major Depressive Disorder and Sleep Disturbances Through Inflammation in Adolescents

**DOI:** 10.3389/fpsyt.2020.559272

**Published:** 2020-09-15

**Authors:** Abhishek Reddy, Mounica Thootkur, Li Li

**Affiliations:** ^1^ Department of Pediatric Pulmonology and Sleep Medicine, University of Alabama at Birmingham, Birmingham, AL, United States; ^2^ Department of Psychiatry and Behavioral Neurobiology, University of Alabama at Birmingham, Birmingham, AL, United States

**Keywords:** adolescents, major depression, sleep disturbance, inflammation, cytokine

## Abstract

**Background:**

Although approximately 13% of adolescents suffer from Major Depressive Disorder (MDD), and many adolescents have reported sleep disturbances, the relationship between sleep disturbances and MDD in adolescents is poorly understood. Thus, our objective was to study how adolescent MDD was related to sleep disturbances in a cross-sectional study, and the potential role of inflammation linking adolescent MDD to sleep disturbances.

**Methods:**

Ninety-two female and male, African American and White, adolescents aged 15 to 18 years completed the study. Adolescents were diagnosed with MDD according to the Diagnostic and Statistical Manual of Mental Disorders-5 as confirmed by the MINI International Diagnostic Interview. The severity of depression was assessed using the Quick Inventory of Depressive Symptomatology. Sleep disturbance was measured using the Pediatric Sleep Questionnaires (PSQ). Blood sample was collected from each participant for measuring the inflammatory factors.

**Results:**

Compared with the controls (n=39), adolescents with MDD (n=53) had greater PSQ scores (0.32 ± 0.02 vs. 0.10 ± 0.02). In adolescents with MDD, PSQ scores were correlated with the severity of depressive symptoms (r=0.31, *p<*0.05). In addition, tumor necrosis factor-α levels were greatly elevated in the MDD group (2.4 ± 0.1 vs. 1.8 ± 0.1 pg/ml) compared with the controls. Severity of depressive symptoms was best predicted by PSQ scores, medications, and childhood experiences.

**Conclusions:**

Sleep disturbance measured by the PSQ is associated with severe depressive symptoms in adolescents, and one potential pathway may be through elevated tumor necrosis factor-α. Further research is warranted to probe a cause and effect relationship among sleep disturbances, MDD, and chronic inflammation.

## Introduction

Recent data by National Institute of Mental Health suggests that an estimated 3.2 million adolescents aged 12 to 17 years in the U.S. have had at least one major depressive disorder (MDD) episode, which represents 13.3% of the U.S. population aged 12 to 17 years. Studies have shown that adolescent depression increases the risk for subsequent depression and anxiety disorders in adulthood (1). Adolescents with MDD are at much higher risk of poor performance at school, using illicit drugs and alcohol, and bingeing (2). Furthermore, depressed adolescents tend to have suicidal ideation (2). In addition, up to 40% to 70% of children and adolescents with MDD also suffer from other psychiatric disorders, and many youngsters have two or more comorbid diagnoses (3). Although the Treatment for Adolescents with Depression Study, a large multi-site clinical research study funded by the NIMH, showed a response rate of 71% on a combination of fluoxetine and cognitive behavioral therapy after 12 weeks, and even 85% response rate at 18 weeks, treatment resistance to both antidepressants and psychotherapy is high in children and adolescents with MDD (4–6). Besides treatment resistance, adherence to treatment is always challenging. It is reported that about 30% to 50% are non-compliant, resulting in poor prognosis and functioning levels in adolescents (7). Thus, high prevalence of MDD in adolescents, limited response rate and high non-compliance indicate that preventive strategies are warranted.

A meta-analysis of longitudinal studies demonstrated that sleep disturbance is one of the primary risk factor for depression (8). It is well-established that one of the core symptoms in adolescent MDD is sleep disturbances, including daytime sleepiness and insomnia (9). The prevalence of insomnia in adolescents is as high as 23.8% (10). Some studies also suggest a significant relationship between sleep disturbances and completed suicide in adolescents (11). Recent studies focusing on sleep architecture using electroencephalogram in depressed adolescents have yielded heterogeneous results. Some studies found less stage 1 and 2 sleep, more sleep awakenings and a shorter latency to rapid eye movement sleep (12). However, majority of studies show no difference in sleep architecture between depressed and non-depressed adolescents (2). In contrast, electroencephalogram sleep findings are much more consistent among different studies in adult depression (12). Therefore, a good understanding of sleep problems in adolescents with MDD is critical.

Prior studies in adults found that chronic inflammation is associated with MDD, and several studies also reported that inflammatory factors are related to the sleep-wake cycle in humans (13–16). Both interleukin (IL)-1β and tumor necrosis factor-α (TNF) were reported to play an important role in the regulation of sleep (17). Similarly, IL-6 was also found to relate with lower sleep efficiency and daytime napping (18, 19). Thus, inflammatory factors may be a link between MDD and sleep disturbances quality in adolescents.

While sleep problem is a core symptom in adolescents with MDD, more studies are needed to precisely understand the relationship between sleep disturbances and MDD in adolescents. Therefore, the current study was designed to determine the relationship between sleep disturbances and adolescent MDD. A secondary aim was to explore the potential pathway by examining the role of inflammatory factors in the relationship between adolescent MDD and sleep disturbances.

## Methods

### Participants

This was a cross-sectional study design. All study procedures were reviewed and approved by the University of Alabama at Birmingham (UAB) Institutional Review Board before enrolling the first participant. Participants were recruited through the community, Birmingham, AL, or the UAB outpatient Psychiatric and Pediatric settings. Study flyers were posted in these areas, and families interested in participation called our office, where study staff explained the study and answered questions. Afterward, interested families and adolescents were invited to come to the laboratory for the enrollment. Both guardians and adolescents provided their written informed consent. Participants included males and females, Whites and African Americans, ages 15 to 18 years and were physically healthy or had stable medical conditions (**Table 1**). Of 99 males and females enrolled in the study, 92 participants completed all study procedures for data analysis. Of the 7 subjects that were excluded, 4 were excluded for sleep disorders, substance use, mania or diabetes, and 3 refused the blood draw. None had evidence of systemic inflammatory diseases or were taking medications known to affect immune system or any antibiotics. Participants with a history or a diagnosis of sleep disorders, including sleep apnea, were excluded. Participants were also excluded from the study if they were pregnant or lactating. Demographic data, medical history, medications, and family information were recorded by self-report. After they were consented, Structured Clinical Interview for DSM-5 or the Mini International Neuropsychiatric Interview for children and adolescents V6.0. was used to diagnose MDD, and administered in each participant (20). Participants who met the criteria for MDD were in the MDD group, and participants who did not meet the criteria for MDD were in the non-MDD or control group. Due to safety concern, adolescents endorsed acute suicidal ideation would be referred for further psychiatric assessment, and would not be enrolled.

**Table 1:** Participant characteristics.

	Control	MDD	*p* values
N	39	53	
Gender, female/male, N	22/17	33/20	0.36
Race, C/AA, N	12/26	40/13	0.00
Age, years	16.1 ± 0.16	16.2 ± 0.13	0.58
QIDS scores	4.7 ± 0.5	13.2 ± 0.8	0.00
Body mass index, kg/m^2^	23.9 ± 1.0	27.8 ± 1.2	0.02
Family income, K	58 ± 9.4	61 ± 7.2	0.80
Childhood trust events scores	2.9 ± 0.4	7.8 ± 0.6	0.00
Guardian education status, ≥ 12 years, N	31	36	0.41
Smoking status, N	2	7	0.17
Antidepressants, N	1	36	0.00

MDD, major depressive disorder; C, Caucasians; AA, African Americans; QIDS, Quick Inventory of Depressive Symptoms.

### Measures

Depressive symptoms in each adolescent were measured using the Quick Inventory of Depressive Symptoms-Adolescent-Self Report (QIDS-A_17_-SR) (21). The scoring system of the QIDS-A_17_-SR comprises 9 domains of depressive symptoms. Each domain weights 0 to 3 and the total score ranges from 0 to 27.

The Pediatric Sleep Questionnaire (PSQ) was used in the current study because it has been extensively studied and shows adequate psychometric properties (22). Scales for PSQ assess for different sleep disturbances, including sleep-related breathing disorder, snoring, daytime sleepiness, and inattentive/hyperactive behavior, and is composed of 22 items that could be completed by their guardians in about 5 min. The 22 items of the scale are each answered yes=1, no=0, or don’t know=missing. The number of items endorsed positively (“yes”) is divided by the number of items answered positively and negatively; the denominator therefore excludes items answered as don’t know (missing responses). Participants in the current study were required to answer all 22 items. The result is a proportion that ranges from 0.0 to 1.0. Score of PSQ greater than 0.33 is suggestive of a diagnosis of sleep-related disorder.

The Childhood Trust Events Survey is a self-report screening survey to assess a child’s exposure to traumatic events (23, 24). It includes 8 categories of adverse events, including physical abuse, emotional abuse, sexual abuse, alcohol exposures, family member in prison, ill caregiver, domestic violence, and loss/separation from caregivers. Each category will receive a score of 1 or 0. If any question in the category is answered yes, then the score for that category will be 1. If all questions in the category are answered no, then the score for that category will be 0. Add all of the numbers in the score column up to receive the total trust events score. Total scores range from 0 to 8, and higher scores reflect less trust.

### Blood Collection and Serum Measurement

Participants presented to the laboratory between 8:00 and 10:00 am, and were not requested to fast before blood collection. Fifteen milliliters of blood were drawn from each participant and centrifuged at 3000*g* for 10 mins. Sera were immediately divided into aliquots, and frozen at −80°C until analysis. The analysis for inflammatory factors, including IL-6, IL-8, IL-10, C-reactive protein (CRP), and TNFα was performed using a Meso Scale Discovery multiplex assay and analyzed with MPSQ Discovery Workbench software (Gaithersburg, MD). Concentrations for IL-6, IL-8, IL-10, and TNFα were expressed in pg/ml, and CRP was expressed in mg/L. All samples were run in duplicate, and the mean of the duplicate samples was reported.

### Statistical Analysis

All analyses were completed with SPSS version 25, and *p* value was set at <0.05 as significant. All variables were tested for normality of distribution by means of Kolmogorov-Smirnoff tests, and would be log-transformed for distribution normality. Data are presented as means ± standard error. Differences between the two groups in variables of interest were compared using Chi-square test for categorical data, including gender, race, smoking status, highest education in guardians, and use of antidepressants and sleeping medications. Independent T-test was used for continuous data, including age, BMI, family income, QIDS scores, childhood trust events scores, PSQ global and subscales scores. Comparisons of PSQ subscales were also conducted after adjusting for race, BMI, antidepressants, and childhood trust events scores. Analysis of covariance (ANCOVA) adjusted for age, race, gender, BMI, childhood trust events scores, and use of antidepressants and sleeping medications was used to determine differences in the inflammatory factors between the two groups. Partial Pearson correlation analysis was used to determine the correlation between sleep disturbances and the severity of depression after controlling for all aforementioned variables in total sample. Mediation analysis was conducted using PROCESS. Stepwise multiple linear regression analysis was used to identify the independent variables that best predicted depression in total sample (n=92). Variables that may contribute to depression were entered into the model, including age, race, gender, PSQ global and subscale scores, BMI, childhood trust events scores, and all measured inflammatory factors.

## Results

### Participants Characteristics

A summary of the 92 participants is presented in **Table 1**. Participants were stratified into MDD (n=53) and non-MDD (controls, n = 39) groups, and there were no significant differences in age, gender, smoking status in adolescents, family income, and guardian’s education status. However, there were more Whites, greater BMI, and childhood trust event scores in the MDD group than in the controls. As expected, depressive scores measured by the QIDS were much greater in the MDD group, and there were 36 adolescents in the MDD group taking antidepressants. Sleep disturbance assessed using the PSQ is reported in **Table 2**. The mean total score on the PSQ was 0.32 ± 0.02 in the MDD group with higher scores indicating sleep disturbances. Additionally, subscales for PSQ were also compared between the two groups. Adolescents with MDD had greater sleep snoring, sleepiness, and hyperactivities in daytime. After controlling for race, BMI, childhood trust event scores, and antidepressants, sleep snoring was not significant although sleepiness and hyperactivities in daytime remained significant between the groups.

**Table 2:** Comparisons of sleep disturbances.

	Control	MDD	*p* values
PSQ scores	0.10 ± 0.02	0.32 ± 0.02	0.001
Snoring	0.64 ± 0.22	1.26 ± 0.19	0.479
Sleepiness	0.49 ± 0.13	2.28 ± 0.20	0.000
Inattentive/hyperactive behavior	0.77 ± 0.20	2.30 ± 0.24	0.010
Sleep medication, N	1	6	0.131

MDD, major depressive disorder; PSQ, pediatric sleep questionnaire.

### Relationship Between Sleep Disturbances and Depression

**Figure 1:**
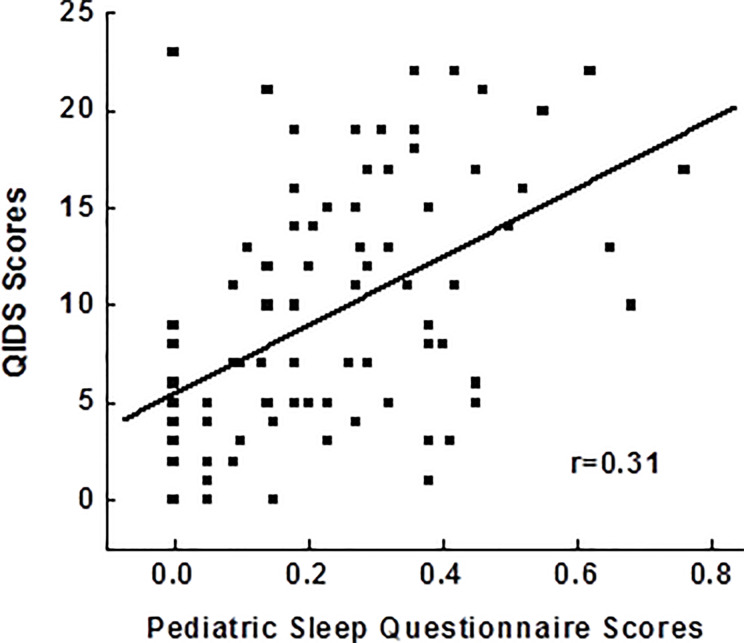
Correlations between pediatric sleep questionnaires and the severity of depressive symptoms. QIDS, Quick Inventory of Depressive Symptoms.

After controlling for age, race, gender, BMI, childhood trust events scores, use of antidepressants and sleeping medications, Pearson correlation analysis in 92 adolescents showed a significant relationship between PSQ scores and depressive scores (*r*=0.31; *p*=0.006), indicating poor disturbance is related to more severe depression (**Figure 1**). PSQ subscales correlation with depressive scores also showed positive relationships with a greatest relationship between hyperactivities on daytime and depression severity (*r*=0.31; *p*=0.005).

### Relationships Between Sleep Disturbances, Depression, and Inflammation

**Figure 2:**
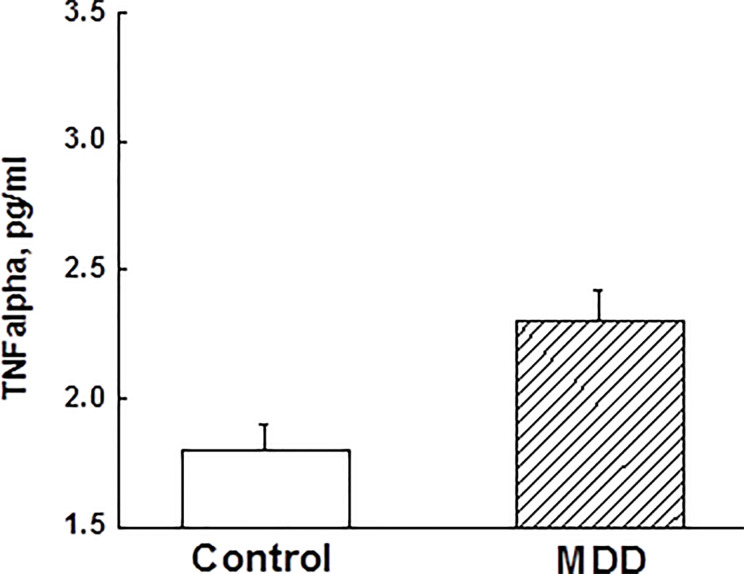
Comparisons of tumor necrosis factor-α between major depressive disorder group and control group in adolescents (*p* < 0.05). TNF, tumor necrosis factor; MDD, major depressive disorder.

ANCOVA adjusting for race, BMI, and childhood trust event scores indicated that adolescent MDD group had significantly elevated TNFα levels compared to the control group (**Figure 2**). However, other measured inflammatory factors did not differ between the 2 groups. Pearson correlation analysis after adjusting for race, BMI, and childhood trust event scores showed significant correlations between PSQ scores and TNFα levels (*r*=0.24; *p*=0.047), as well as hyperactivities and TNFα levels (*r*=0.34; *p*=0.004). Mediation analysis showed that TNFα had an indirect effect of 12.4% on the link of MDD with sleep disturbance in adolescents. Stepwise multiple linear regression analysis indicated that the severity of depressive symptoms was best predicted by medications, PSQ scores, and childhood trust event scores (**Table 3**).

**Table 3:** Stepwise linear regression analysis predicting depression.

Dependent variable	Significant predictors	B	R^2^
QIDS scores	PSQ scores	5.83	0.44
QIDS scores	PSQ scores, medications	4.48	0.49
QIDS scores	PSQ scores, medications, childhood trust event scores	3.36	0.53

Independent variables included age, race, gender, body mass index, smoking status, PSQ scores, measured inflammatory factors. QIDS, quick inventory of depressive symptoms; PSQ, pediatric sleep questionnaire.

## Discussion

In this study we explored the relationship between sleep disturbances and MDD in adolescents, as well as a potential underlying pathway by studying the role of chronic inflammation. Our study clearly shows that sleep disturbance is associated with MDD in adolescents. Our data further suggests that as the sleep disturbance gets greater, the symptoms of MDD get worse. Mechanistic study revealed that TNFα was significantly elevated in adolescent MDD compared to controls, positively associated with PSQ scores, and play an indirect role in the association between MDD and sleep disturbance. To our knowledge, this is the first study to explore the relation between sleep disturbances, MDD, and the role of inflammation in adolescents.

Recent literature shows increasing evidence of relationship between sleep disturbance and MDD in adolescents (2). Literature review also suggests sleep disturbance in adolescents is likely to predict depression (2). Studies show that adolescents with depression have problems with sleep-onset, being unable to return to sleep after waking prematurely, and hypersomnia (25, 26). There is strong evidence of consequences of sleep disturbances in adolescents including depression, anxiety, increased risk of suicidal ideation, and also increased risk of obesity (27). In our study, we showed that adolescents with MDD scored much higher in PSQ scale with snoring, sleepiness, and increased hyperactivity during the day. Thus, our findings are consistent with the literature that not only there is sleep disturbances at night in adolescents who have depression but also there is functional impairment during the day (18, 28). The association of sleep disturbances with MDD also indicates that early and adequate treatment of sleep disturbances, including insomnia, might contribute to the treatment for MDD in adolescents. The relationship between all the above has not been well studied in well-designed longitudinal studies utilizing sleep-focused therapy to explore the impact of addressing sleep disturbances on MDD, and is an area that needs further investigation.

Our previous study and many other studies have shown that adults with MDD show increased circulating levels of inflammatory markers like IL-6 and TNFα (13, 29). Pro-inflammatory cytokines like TNFα can induce indoleamine 2-3-dioxygenase, an enzyme that results in catabolism of tryptophan resulting in decreased serotonin levels. In addition, TNFα can also influence the hypothalamic-pituitary-adrenal axis by increasing corticotrophin releasing hormone release and disturbing the function of the glucocorticoid receptor (30). Thus, there is evidence in different studies of TNFα’s role in depression, and we postulate a similar role for TNFα in relation to MDD in adolescents as evidenced by elevated levels in our study.

As compared to inflammatory markers in adults with MDD, there is limited data with significant variation in inflammatory markers in adolescents with MDD (31, 32). The small number of studies and sparse data could be one of the main reasons preventing researchers from reaching a conclusion on the role of inflammatory markers in adolescent depression. In addition, TNFα levels were compared only in adolescents with MDD and suicidal ideation, which also showed variations (31). Unique to our study is the fact that we looked at adolescents with only MDD and no suicidal ideation, and they had significantly elevated TNFα levels compared to the control group which is consistent with findings in multiple adult studies (29). However, other inflammatory markers, including IL-6, IL-8, IL-10, and CRP, did now show significant differences between controls and MDD group in our study. Numerous factors have been postulated causing the differences in inflammatory markers in adolescent MDD and adult MDD. Some of them have been attributed to changes in neuronal development, hormonal changes, stress, trauma, and differences in immunity in adults as compared with adolescents (31).

There is strong evidence in literature that TNFα plays an important role in sleep regulation in both animal and human studies (15, 17). In pediatric obstructive sleep apnea patients, TNFα levels increase, and are closely related to sleepiness (33). Also, TNFα levels and sleepiness in these patients decreased after surgery (33). In healthy young men, during sleep, plasma TNFα decreases (34). Consistently, in humans with sleep deprivation, circulating levels of TNF-receptor increase (35). Thus, TNF system may play an important role in normal circadian regulation (14). In the present study, we found increased TNFα levels in adolescents with MDD who displayed sleep disturbances. Thus we explore the possibility of interplay of sleep disturbances and TNFα in MDD in adolescents which has not been explored before.

There are a few limitations that are needed to note when our results are interpreted. Frist, our sample size is relatively small, though it is still bigger than some of previous studies for inflammatory markers in adolescents. Second, it is a cross-sectional study design, so a causal-effect relationship could not be established. Third, the assessment of sleep disturbance was based on a validated but self‐reported questionnaire, which has been widely used in clinical research when polysomnography is not feasible (22). However, there may not be perfect overlap between self-reported questionnaires and objective measures from polysomnography. Finally, more studies are needed to explore the role of other inflammatory markers in adolescents with MDD and sleep disturbances. However, our findings indicate that timely treatment of sleep problems in adolescents will help address MDD earlier and help with academic performance, prevent drug abuse and help with inter personal relationships (2). Our paper brings attention to the possibility of treating MDD in adolescents by addressing both sleep and inflammation at the same time.

## Data Availability Statement

The raw data supporting the conclusions of this article will be made available by the authors following the National Institutes of Health guidelines.

## Ethics Statement

All study procedures were reviewed and approved by the University of Alabama at Birmingham (UAB) Institutional Review Board before enrolling the ﬁrst participant. Written informed consent to participate in this study was provided by the participants’ legal guardian.

## Author Contributions

AR analyzed the data, drafted, edited, and approved the final manuscript. MT drafted, edited, and approved the final manuscript. LL designed and performed the study, analyzed the data, and edited and approved the final manuscript.

## Funding

This research was supported by awards, P30DK056336 and P30DK079626, from the National Institute of Diabetes and Digestive and Kidney Diseases to Nutrition Obesity Research Center and Diabetes Research Center, respectively, at the University of Alabama at Birmingham.

## Conflict of Interest

The authors declare that the research was conducted in the absence of any commercial or financial relationships that could be construed as a potential conflict of interest.

## Abbreviations

MDD, major depressive disorder; TNF, tumor necrosis factor; DSM-5, Diagnostic and Statistical Manual of Mental Disorders-5; PSQ, Pediatric Sleep Questionnaire; IL, interleukin; QIDS-A_17_-SR, Quick Inventory of Depressive Symptoms-Adolescent-Self Report; ANCOVA, analysis of covariance; BMI, body mass index.
